# Graft-Versus-Host Disease–Free Antitumoral Signature After Allogeneic Donor Lymphocyte Injection Identified by Proteomics and Systems Biology

**DOI:** 10.1200/PO.18.00365

**Published:** 2019-05-23

**Authors:** Xiaowen Liu, Zongliang Yue, Yimou Cao, Lauren Taylor, Qing Zhang, Sung W. Choi, Samir Hanash, Sawa Ito, Jake Y. Chen, Huanmei Wu, Sophie Paczesny

**Affiliations:** ^1^ Indiana University School of Informatics and Computing, Indianapolis, IN; ^2^ Indiana University School of Medicine, Indianapolis, IN; ^3^ University of Alabama at Birmingham School of Medicine, Birmingham, AL; ^4^ Fred Hutchinson Cancer Research Center, Seattle, WA; ^5^ University of Michigan, Ann Arbor, MI; ^6^ MD Anderson Cancer Center, Houston, TX; ^7^ National Heart, Lung, and Blood Institute, Bethesda, MD

## Abstract

**PURPOSE:**

As a tumor immunotherapy, allogeneic hematopoietic cell transplantation with subsequent donor lymphocyte injection (DLI) aims to induce the graft-versus-tumor (GVT) effect but often also leads to acute graft-versus-host disease (GVHD). Plasma tests that can predict the likelihood of GVT without GVHD are still needed.

**PATIENTS AND METHODS:**

We first used an intact-protein analysis system to profile the plasma proteome post-DLI of patients who experienced GVT and acute GVHD for comparison with the proteome of patients who experienced GVT without GVHD in a training set. Our novel six-step systems biology analysis involved removing common proteins and GVHD-specific proteins, creating a protein-protein interaction network, calculating relevance and penalty scores, and visualizing candidate biomarkers in gene networks. We then performed a second proteomics experiment in a validation set of patients who experienced GVT without acute GVHD after DLI for comparison with the proteome of patients before DLI. We next combined the two experiments to define a biologically relevant signature of GVT without GVHD. An independent experiment with single-cell profiling in tumor antigen–activated T cells from a patient with post–hematopoietic cell transplantation relapse was performed.

**RESULTS:**

The approach provided a list of 46 proteins in the training set, and 30 proteins in the validation set were associated with GVT without GVHD. The combination of the two experiments defined a unique 61-protein signature of GVT without GVHD. Finally, the single-cell profiling in activated T cells found 43 of the 61 genes. Novel markers, such as RPL23, ILF2, CD58, and CRTAM, were identified and could be extended to other antitumoral responses.

**CONCLUSION:**

Our multiomic analysis provides, to our knowledge, the first human plasma signature for GVT without GVHD. Risk stratification on the basis of this signature would allow for customized treatment plans.

## INTRODUCTION

Allogeneic hematopoietic cell transplantation (HCT) is one of the most effective forms of tumor immunotherapy available to date. The lymphocytes in the donor graft recognize and eliminate residual tumoral cells through the graft-versus-tumor (GVT) effect, and thus, donor lymphocyte injection (DLI) often is used at the time of relapse post-HCT to induce GVT. However, GVT can occur in parallel with lymphocyte reactivity to normal host tissues, which gives rise to graft-versus-host disease (GVHD). Despite the correlation of GVT and GVHD, indirect evidence for a GVT reaction separate from GVHD has been reported in large cohorts of HCT patients or when DLI is administered to induce remission in HCT patients who have experienced a relapse.^1,2^ GVT activity can be increased by targeted therapy, as has been shown with sorafenib in FLT3 internal tandem duplication–mutant leukemia cells.^3^

The GVT effect is mediated by minor histocompatibility antigens (miHAgs) on recipient leukemic cells that are recognized by donor CD4^+^ and CD8^+^ T cells.^4^ miHAgs can mediate GVT without inducing GVHD if they are only expressed by the recipient hematopoietic cells (ie, minor H antigen [HA]-1, HA-2, BCL2A1, and HB-1).^5,6^ Nonhistocompatibility antigen proteins expressed by tumor cells, called tumor-associated antigens (survivin, Wilms tumor 1, proteinase 3) also can mediate GVT activity.^7^ Cytokines (ie, interleukin-15 [IL-15])^3^; checkpoint proteins, such as PD-1/PD-L1^8^; and T-cell trafficking modulators^9^ are other possible mediators of GVT.

Our study aim was to develop a proteomic signature to identify GVT without GVHD after allogeneic DLI. We used an intact-protein analysis system (IPAS) coupled with protein tagging as previously reported by us^10^ first in a training set and then in a validation set and added a novel systems biology pipeline to identify a signature of 61 proteins that are significantly expressed in the plasma of HCT patients who received DLI for tumor relapse. Furthermore, 43 (70%) of the 61 genes were found in tumor antigen (PRAME)–activated T cells from a patient with post-HCT relapse.

**CONTEXT SUMMARY**

**Key Objective**
Separating graft-versus-host disease (GVHD) from graft-versus-tumor (GVT) effect after hematopoietic cell transplantation (HCT) has proven difficult. We attempted to do so through plasma proteomics and systems biology analyses of patients in relapse after HCT who received donor lymphocytes as immunotherapy.
**Knowledge Generated**
Our novel six-step systems biology analysis involved the removal of common proteins and GVHD-specific proteins, creation of a protein-protein interaction network, calculation of relevance and penalty scores, and visualization of candidate biomarkers in gene networks to define a unique, biologically relevant 61-protein signature of GVHD-free GVT. Forty-three of the 61 genes also were found in an independent experiment using massive single-cell profiling of tumor antigen–activated T cells from a patient who experienced post-HCT relapse.
**Relevance**
This multiomic analysis provides the first human plasma signature for GVHD-free GVT to our knowledge. Novel, biologically relevant markers were identified and could be extended to other antitumoral responses. Risk stratification on the basis of the GVT without GVHD protein signature would allow for customized treatment plans after HCT.

## PATIENTS AND METHODS

All patients or their legal guardians provided written informed consent to participate in this study, and the collection of samples for studying post-HCT complications was approved by the institutional review board of the University of Michigan. The methodology for this study is described in the Data Supplement.

## RESULTS

### Plasma Proteomics of GVHD-Free GVT in a Training Set

**FIG 1.**
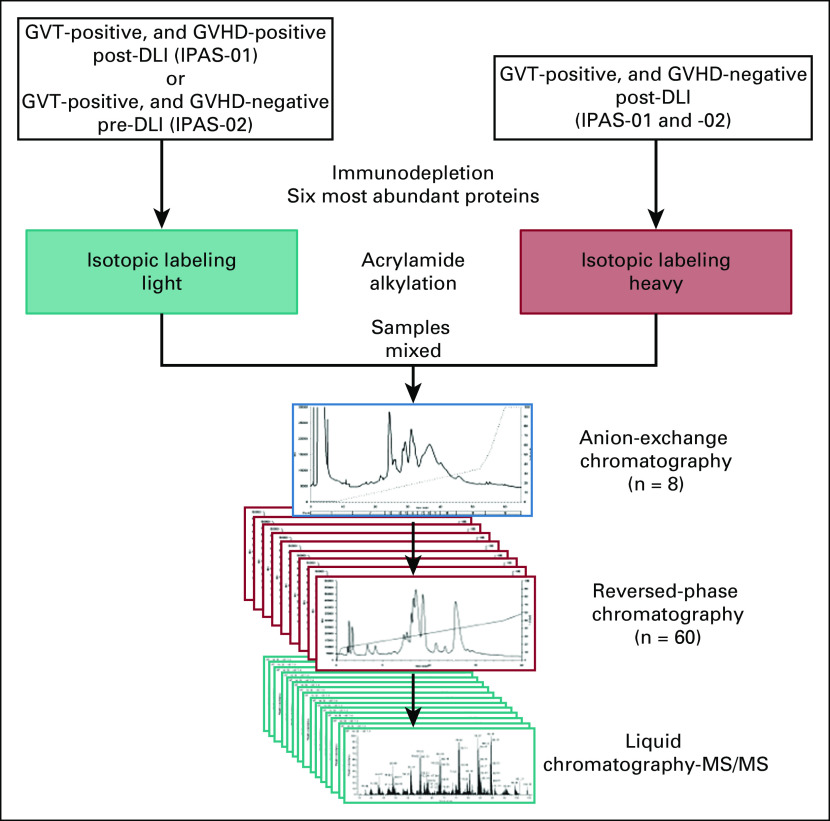
Pool selection and top-down tandem mass spectrometry (MS/MS) workflow to keep intact proteins. The intact-protein analysis system (IPAS) compared graft-versus-tumor (GVT)–positive and graft-versus-host disease (GVHD)–negative post–donor lymphocyte injection (DLI; heavy isotope) with GVT-positive and GVHD-positive post-DLI (light isotope) samples in a training set (IPAS-01), and GVT-positive and GVHD-negative post-DLI (heavy isotope) with GVT-positive and GVHD-negative pre-DLI (light isotope) samples in a validation set (IPAS-02).

Our initial approach to identifying GVT-specific biomarkers was to undertake a proteomics analysis. We hypothesized that proteomes of GVT are distinct from those of GVHD. We reasoned that the best setting to observe a GVT effect without any effect of chemotherapy or preparative conditioning regimen would be after DLI is given for relapse of malignant disease. Our first proteomics experiment (IPAS-01) was designed to distinguish proteomes that predict GVHD-free GVT from those that predict GVT with GVHD after DLI in a training set. We compared a pool of plasma samples taken approximately 30 days after DLI from five patients without relapse and without GVHD (GVT-positive, GVHD-negative) labeled with the heavy isotope and compared them with a pool of plasma samples at matched time points from 11 patients without relapse but with concomitant GVHD (GVT-positive, GVHD-positive) labeled with the light isotope (Fig 1). In this design, only proteins with the heavy/light isotope ratio greater than 1.2 (upregulated) will be GVHD free and considered for additional analyses. The patient and DLI characteristics are listed in the Data Supplement. No significant differences between groups were observed for patient age, disease status (all but one with morphologic relapse, and only one patient did not receive chemotherapy), DLI donor type, and DLI dose, and all patients were off immunosuppression. All patients were evaluated for remission using morphologic evaluation.

A total of 825 proteins were confidently identified and quantified using the SwissProt database (Data Supplement). Of these proteins, 218 were upregulated with a heavy/light ratio greater than 1.2 in GVT-positive, GVHD-negative samples (and either not identified in GVT-positive, GVHD-positive samples or identified with a ratio 1.2 or less). Similar results were obtained using the UniProt human proteome database (Data Supplement). A total of 755 proteins were identified, including 327 that were upregulated by more than 1.2-fold in GVT-positive, GVHD-negative samples. The threshold of 1.2-fold also was used in our most recent GVHD clinical proteomics, which has allowed the discovery of biologically relevant biomarkers.^11^

**FIG 2.**
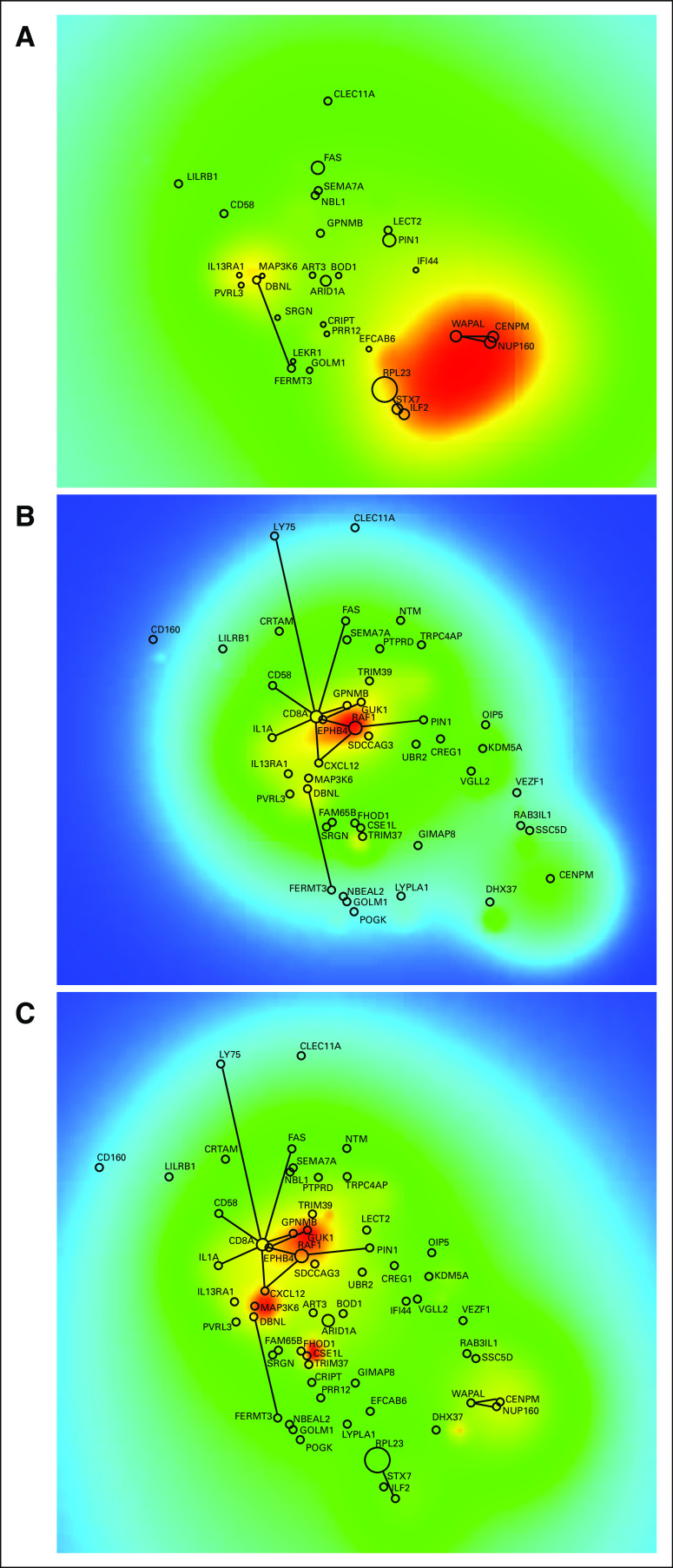
Biomarker selection through GeneTerrain visualization tools shown in a two-dimensional (2D) panel. (A) Intact-protein analysis system (IPAS)-01: GeneTerrain visualization shown in 2D for the graft-versus-tumor (GVT) signature obtained by comparing GVT-positive, graft-versus-host disease (GVHD)–negative with GVT-positive, GVHD-positive post–donor lymphocyte injection (DLI) samples. The nodes between genes represent the protein-protein interaction pairs from the STRING (Search Tool for the Retrieval of Interacting Genes/Proteins) database. The protein-protein interaction confidence score in STRING contains functional protein associations derived from in-house predictions and homology transfers as well as from several externally maintained databases. Each interaction is assigned a score between 0 and 1, which is meant to be the probability that the interaction really exists given the available evidence. (B) IPAS-02: GeneTerrain visualization shown in 2D for the GVT signature from the pre- and post-DLI comparison. (C) Combined IPAS-01 and IPAS-02: GeneTerrain visualization shown in 2D for GVT without GVHD from both analyses.

Because the ratio-based approach is imperfect and can still capture some GVHD proteins, we performed additional steps in the analysis to remove common proteins and GVHD-specific proteins as well as to enrich for biologically relevant proteins with our novel systems biology analysis briefly described in the next sentence and in detail in the Data Supplement. Our computational biology approach involved six processing steps that were modified from our previous workflow^12,13^ to fit the GVT experiment and includes generating abundance ratios from comparisons of plasma samples taken from GVT without GVHD, filtering out GVHD-specific proteins identified in previous experiments, generating protein-protein interaction (PPI) pairs with and without outer gene networks, selecting GVT-relevant proteins by relevance scores and penalty scores for unspecific proteins found in other diseases, and using a visual analytic software tool (Data Supplement). After this data processing, the IPAS-01 experiment provided a list of 46 proteins associated with a GVT signature without GVHD post-DLI in the training set (Table 1). Although we started with IPAS proteins with a heavy/light ratio greater than 1.2, the systems biology process may have resulted in a final score of less than 1; however, because all these proteins are GVT specific, they all are included in the final GVHD-free GVT signature. By using the integrated relevant interaction network as the base layout and differentially expressed proteins in the candidate protein list as response variables, we constructed a GeneTerrain (www.terrainatlas.com) visualization as shown in Figure 2A. An overview of the GeneTerrain object representation is shown in the Data Supplement. Briefly, the terrain base is a nodes-weighted graph that incorporates PPI networks. The *z*-axis adds data obtained from the proteomics experiments to generate a 2-dimensional terrain heat map. The first node is centered around CD8-α (CD8A) and includes CD58, IL1A, Ly75, FAS, and GPNMB and a secondary node centered around RAF1 and containing GUK1, EPHB4, and CXCL12. For example, CD8A is expressed on the surface of most cytotoxic CD8^+^ T lymphocytes and is a coreceptor of the T-cell receptor. The relative weight of CD4^+^ and CD8^+^ T cells in GVHD and GVT responses remains undetermined. Our data with the CD8A-centered node strongly suggest that GVT is majorly mediated by CD8 T cells and GVHD less so, which provides an opportunity to separate GVT and GVHD by manipulating these two T-cell subsets differently. A second cluster comprises MAP3K6, DBNL, and FERMT3, which are all implicated in immune response pathway activity. Penalty scores are designed to enrich for the most relevant proteins without altering the primary data. As a proof of concept, we performed the experiment without applying these proteins to show that their final scores have not been significantly changed by the use of penalty scores (Table 1; Data Supplement).

**TABLE 1.**
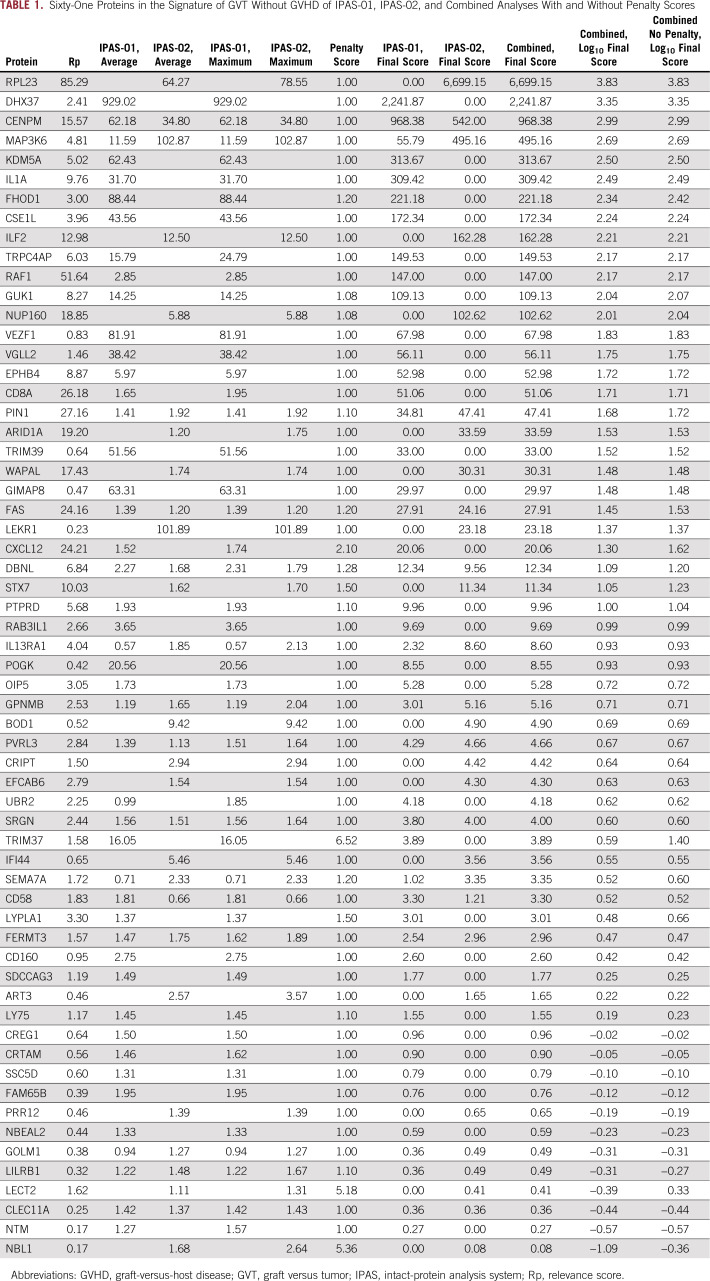
Sixty-One Proteins in the Signature of GVT Without GVHD of IPAS-01, IPAS-02, and Combined Analyses With and Without Penalty Scores

### Plasma Proteomics of GVHD-Free GVT in a Validation Set

Our second proteomics experiment (IPAS-02) used a validation set that was designed to compare a pool of samples from five GVT-positive, GVHD-negative (labeled with the heavy isotope) patients with a pool of samples from the same patients taken before DLI when the patients were in relapse and thus GVT negative (labeled with the light isotope; Fig 1). A total of 733 proteins were identified and quantified using the SwissProt database (Data Supplement). Of these proteins, 125 were upregulated by more than 1.2-fold in GVT-positive, GVHD-negative post-DLI samples (and either not identified in GVT-positive, GVHD-negative pre-DLI samples or identified with a ratio of 1.2 or less). Similar results were obtained using the UniProt human proteome database (Data Supplement). A total of 602 proteins were identified, including 274 proteins that were upregulated by more than 1.2-fold in GVT-positive, GVHD-negative samples.

We then applied the same systems biology workflow as in the training set and obtained 30 proteins associated with a GVHD-free GVT signature from the pre- and post-DLI comparison in the validation set (Table 1). The GeneTerrain visualization is shown in Figure 2B. A major cluster is constituted by centromere protein M (CENPM), NUP160, WAPAL, DHX37, STX7, and IL enhancer binding factor 2 (ILF2), which are all proteins implicated in the activation of cytotoxic T cells consistent with a GVT response (Table 2). For example, CENPM (also called PANE1) is a known miHAg expressed on B-lymphoid cells that is highly relevant to GVT-mediated post-DLI.^12^ The protein encoded by ILF2 is a transcription factor needed for T-cell expression of IL-2.^13^ The second cluster is similar to the one found in IPAS-01 centered on MAP3K6 and DBNL.

**TABLE 2.**
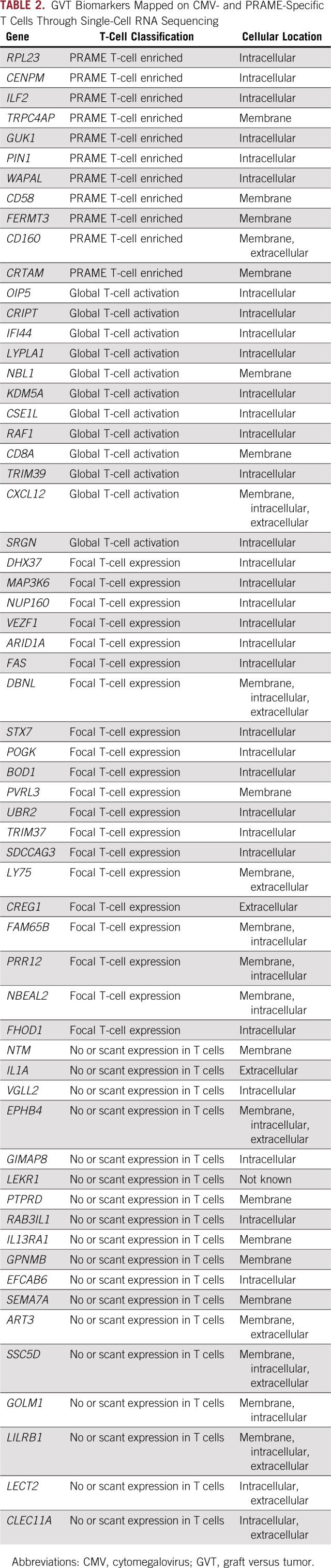
GVT Biomarkers Mapped on CMV- and PRAME-Specific T Cells Through Single-Cell RNA Sequencing

### Final 61-Protein GVHD-Free GVT Signature After DLI

The next step was to combine the proteins found in the training and validation sets. Combined analysis of the two IPAS experiments yielded 61 proteins: 49 with a final combined score of greater than 1 and 12 with a final combined score of less than 1, all specific to GVT without GVHD (Table 1). The GeneTerrain visualization is shown in Figure 2C. Four main clusters are seen. These include one similar to that seen in IPAS-01 centered on CD8A, one similar to that seen in IPAS-02 centered on CENPM-WAPAL and also containing DHX37, and one found in both experiments and centered on MAP3K6 and DBNL. A new fourth cluster appeared as a result of the enrichment in proteins specific for GVT without GVHD by the combination of the two sets. This additional cluster is centered around CSE1L, which is a RAS-related nuclear protein with a potential role in RAS/RAF/MAPK signaling in T cells. Some potential important novel markers, such as cytotoxic and regulatory T-cell molecule (CRTAM), which has been shown to determine the CD4^+^ cytotoxic T-lymphocyte lineage,^14^ are not part of a cluster. A simple explanation is that CRTAM has been discovered recently, so the literature on CRTAM is only starting to emerge, which means that databases, such as STRING (Search Tool for the Retrieval of Interacting Genes/Proteins), have not yet integrated PPI networks for this protein.

### Forty-Three of 61 Markers Are Found in a Single-Cell RNA Sequencing Analysis of PRAME- and Cytomegalovirus-Specific T Cells Post-HCT Relapse

**FIG 3.**
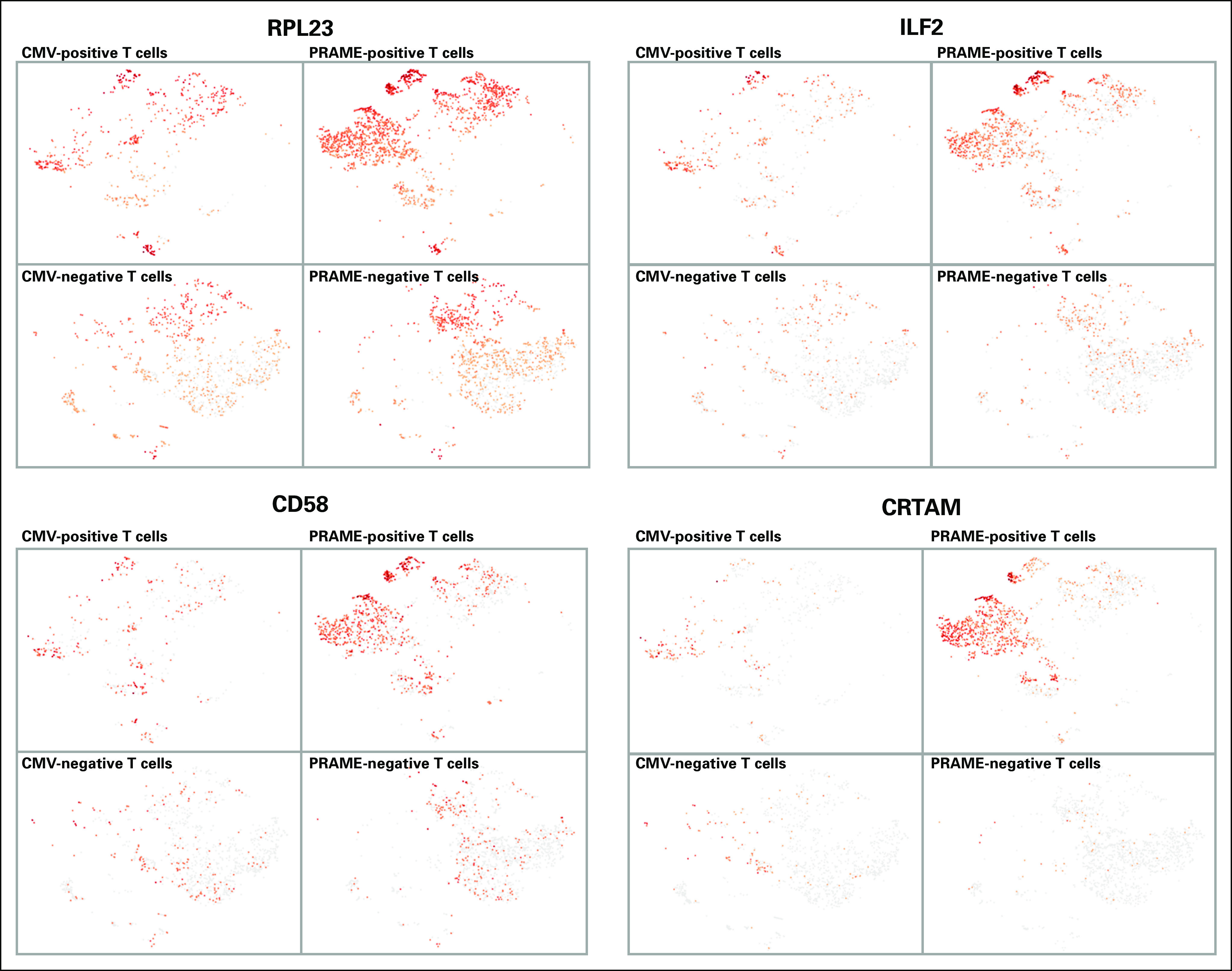
Expression profile of four representative graft-versus-tumor (GVT) markers in single-cell RNA sequencing analysis of PRAME- and cytomegalovirus (CMV) pp65–specific T cells. Expression profiles are shown for representative GVT markers RPL23, ILF2, CD58, and CRTAM in CMVpp65-positive or -negative T cells and PRAME-positive or -negative T cells.

Because the signature contained mostly intracellular proteins and additional samples from the discovery sets were not available to us, we next used single-cell profiling on T cells sorted and stimulated by the tumor antigen PRAME from a patient who relapsed after HCT compared with T cells sorted and stimulated by the viral antigen cytomegalovirus (CMV) pp65a as a surrogate measurement for an antitumoral response post-HCT. A mean reads number of 381,079 per cell and median gene number of 1,091 per cell were analyzed for an average of 1,436 cells per condition. Among the 61 genes identified in the proteomic GVHD-free GVT signature, 11 were more highly expressed in PRAME-specific T cells compared with CMV-specific T cells or nonreactive T cells. Thirty-two genes were enriched in both CMV- and PRAME-specific T cells, which represent a general activation marker of T cells. In contrast, 18 genes were not expressed in T-cell populations. The function and main GeneGO (Clarivate, Philadelphia, PA) process for each protein are described in Table 2. The expression profile of four representative GVT markers in the single-cell RNA sequencing analysis of PRAME-specific T cells (RPL23, ILF2, CD58, and CRTAM) is shown in Figure 3. RPL23 is a component of the 60S ribosomal subunit and has been shown to link the oncogenic RAS signaling to p53-mediated tumor suppression.^15^ T-cell responses to RPL23 also are increased in autoimmune diseases,^16^ which suggests a role for the RAS/RAF/MAPK pathway in T cells that is currently underexplored. CD58 is the ligand of CD2 on T cells, and engagement of both lead to T-cell activation and adhesion. The CD58/CD2 axis is the primary costimulatory pathway for CD8^+^ T cells that lack CD28, which suggests an alternate activation mechanism during GVT for exhausted T cells.^17^ CRTAM is upregulated in CD4^+^ and CD8^+^ T cells and encodes a type 1 transmembrane protein with V and C1-like immunoglobulin domains.^18^ It has been shown to negatively regulate ZEB1 (zinc finger E-box–binding homeobox 1) in T cells^19^ and to determine the CD4^+^ cytotoxic T-lymphocyte lineage,^14^ and it might determine the CD8^+^ cytotoxic T-lymphocyte lineage as well. The expression profiles of all 61 GVHD-free GVT markers are shown in the Data Supplement. These results suggest that tumor-specific T cells serve as a source of cells that express the GVT signature but require additional validation in future studies.

## DISCUSSION

The current study has identified the first plasma proteomic signature for GVHD-free GVT after DLI using an in-depth tandem mass spectrometry–based analysis of plasma combined with a novel six-step systems biology approach. Previous studies have focused on cellular T-cell markers.^20^ This pipeline allowed us to identify a final plasma signature of 61 proteins from initially thousands of proteins. One systems biology novelty of our approach was the performance of a one-layer extension on PPI data using interactions one node away from the gene in the original list that are called outer genes. However, we found that this strategy did not significantly change the final signature of 61 proteins (data not shown). A strength of our approach is that we filtered out nonrelevant proteins using a penalty score, which led to a more-specific list of candidate proteins and avoided contaminants. This study favored a large-scale proteomics approach as opposed to a hypothesis-driven candidate approach.^21^ We have shown that for GVHD markers, this method is efficient in discovering new candidate markers.^10,11^ Compared with our previous studies, we experimentally removed the GVHD proteins by assigning them the light isotope. Of note, this approach showed that the proteins identified and their ratio have not been influenced much by the implementation of penalty scores. In an independent experiment with single-cell profiling of T cells from a patient with relapse after HCT that were activated in vitro, 43 of the 61 proteomic genes also were found in activated T cells, which suggests that the proteins identified are biologically relevant in different antitumoral responses.

The biology of the GVT markers not yet described is as follows. The function of TRPC4AP (transient receptor potential cation channel subfamily C member 4–associated protein) has been shown to be involved in the ubiquitination of E3 ligase skp2^22^ and the activation of c-Jun NH(2) terminal kinase and transcription factor AP-1.^23^ Guanylate kinase 1 (GUK1) is an enzyme that catalyzes the transfer of a phosphate group from ATP to guanosine monophosphate (GMP) to form guanosine diphosphate (GDP) and is believed to be a good target for cancer chemotherapy.^24^ Its expression on tumor-specific T cells was not previously reported. PIN1 (peptidylprolyl *cis*/*trans* isomerase, NIMA-interacting 1) catalyzes the *cis*/*trans* isomerization of peptidyl-prolyl peptide bonds and thus catalytically regulates the postphosphorylation conformation of its substrates and is involved in the regulation of T-cell biology. In particular, its implication has been shown in systemic lupus erythematosus and T-cell acute lymphoblastic leukemia progression.^25-27^ WAPL cohesin release factor has been shown to restrict chromatin loop extension.^28^ Of note, it was part of a microRNA-mRNA network in allogeneic T-cell responses.^29^ Fermitin family member 3 (FERMT3) is a member of the kindlins that mediates PPI involved in integrin activation. Mutations in this gene cause the autosomal recessive leukocyte adhesion deficiency syndrome-3.^30^ Its role in T cells has not been studied. CD160 is another surface protein tightly expressed on peripheral cytotoxic CD8 T lymphocytes and natural killer cells,^31^ and soluble CD160 enhances CD8^+^ T cells, which results in increased interferon-γ, IL-2, and tumor necrosis factor-α secretion as well as cytolysis against tumor cells in vitro and in vivo.^32^

Not surprisingly, serglycin (SRGN), which serves as a mediator of granule-mediated apoptosis through the macromolecular complex of granzymes and perforin and determines the secretory granule repertoire of cytotoxic T lymphocytes,^33^ was also upregulated in this study. The Fas cell surface death receptor (FAS) that plays a critical role in the activation of the death-inducing signaling complex with Fas-associated death domain protein and triggers a downstream caspase cascade that leads to apoptosis^34^ also was upregulated.

The chemokine CXCL12 has been proposed to be able to distinguish immune cells that induce GVT going to the bone marrow from immune cells that induce GVHD.^35^ It is one of the rare proteins for which the final score was modified more than twice by the penalty score but is still included overall as a GVT protein. Several of the tripartite motif (TRIM) members encode for miHAgs.^21^ Although TRIM42, 22, and 37 are located on chromosomes 3, 11, and 17, respectively, TRIM39 is on chromosome 6 in the major histocompatability class I region and, in our study, showed global expression on activated T cells. RAF1 is the cellular homolog of viral raf proto-oncogene (v-raf) and is also a MAP3K that functions downstream of the Ras family of membrane-associated GTPases to which it binds directly. CSE1L is a RAS-related nuclear protein that binds strongly to nuclear localization signal-free importin-α, and this complex is then released in the cytoplasm by the combined action of RANBP1 and RANGAP1. The role of RAS/RAF/MAPK and phosphatidylinositol 3-kinase signaling in T cells remains understudied. One particular protein of non–T-cell origin, IL-1α, was found to be elevated in the plasma of patients with GVT response and may play a significant role in antitumor effects. Of note, IL-1α–engineered tumor cells rarely develop into tumors, and if they do, the tumors are quickly destroyed through a mechanism that involves CD8^+^ T cells and natural killer cells, and IL-1α enhances immunoediting in the tumor microenvironment.^36^

Although our approach identified several proteins for GVHD-free GVT, there are limitations in this study. First, plasma samples were pooled together. Second, only a small percentage of the whole circulating proteins are identified and quantified with tandem mass spectrometry. Third, the systems biology pipeline relies on knowledge from the published domain, which makes the application of this method to diseases that have not been well studied, such as GVT, difficult. Finally, because most of the proteins identified were intracellular, we could not apply immunoassays as we have done previously. As a result of the lack of samples available from additional DLI patients, we sought to look at the 61 genes from the proteomic signature in T cells from a patient who experienced relapse after HCT and that were stimulated by the tumor antigen PRAME as a surrogate measurement for an antitumoral response post-HCT. Forty-three of these 61 genes also were found with this technique. Although challenged by the paucity of samples available after DLI, validation of this proteomic signature in larger cohorts is warranted.
